# Correlation of postoperative fluid balance and weight and their impact on outcomes

**DOI:** 10.1007/s00423-020-02004-9

**Published:** 2020-10-13

**Authors:** Fabio Butti, Basile Pache, Michael Winiker, Fabian Grass, Nicolas Demartines, Martin Hübner

**Affiliations:** 1 Department of Visceral Surgery, Lausanne University Hospital CHUV, Rue du Bugnon 46, CH – 1011 Lausanne, Switzerland; 2 Department of General Surgery, GHOL Nyon Hospital, Ch. Monastier 10, 1260 Nyon, Switzerland; 3 Department of Gynaecology and Obstetrics, Lausanne University Hospital CHUV, Rue du Bugnon 46, 1011 Lausanne, Switzerland; 4 Department of General Surgery, HRC Rennaz Hospital, Route du Vieux Séquoia 20, 1847 Rennaz, Switzerland

**Keywords:** Fluid balance, Weight gain, Complications, Outcome, Colorectal

## Abstract

**Introduction:**

Normovolemia after major surgery is critical to avoid complications. The aim of the present study was to analyze correlation between fluid balance, weight gain, and postoperative outcomes.

**Methods:**

All consecutive patients undergoing elective or emergency major abdominal surgery needing intermediate care unit (IMC) admission from September 2017 to January 2018 were included. Postoperative fluid balances and daily weight changes were calculated for postoperative days (PODs) 0–3. Risk factors for postoperative complications (30-day Clavien) and prolonged length of IMC and hospital stay were identified through uni- and multinominal logistic regression.

**Results:**

One hundred eleven patients were included, of which 55% stayed in IMC beyond POD 1. Overall, 67% experienced any complication, while 30% presented a major complication (Clavien ≥ III). For the entire cohort, median cumulative fluid balance at the end of PODs 0–1–2–3 was 1850 (IQR 1020–2540) mL, 2890 (IQR 1610–4000) mL, 3890 (IQR 2570–5380) mL, and 4000 (IQR 1890–5760) mL respectively, and median weight gain was 2.2 (IQR 0.3–4.3) kg, 3 (1.5–4.7) kg, and 3.9 (2.5–5.4) kg, respectively. Fluid balance and weight course showed no significant correlation (*r* = 0.214, *p* = 0.19). Extent of surgery, analyzed through Δ albumin and duration of surgery, significantly correlated with POD 2 fluid balances (*p* = 0.04, *p* = 0.006, respectively), as did POD 3 weight gain (*p* = 0.042). Prolonged IMC stay of ≥ 3 days was related to weight gain ≥ 3 kg at POD 2 (OR 2.8, 95% CI 1.01–8.9, *p* = 0.049).

**Conclusion:**

Fluid balance and weight course showed only modest correlation. POD 2 weight may represent an easy and pragmatic tool to optimize fluid management and help to prevent fluid-related postoperative complications.

**Electronic supplementary material:**

The online version of this article (10.1007/s00423-020-02004-9) contains supplementary material, which is available to authorized users.

## Introduction

Fluid management standards in patients undergoing major surgery have changed in the last years. Several studies revealed a direct relationship between perioperative fluid balance and postoperative adverse events [1–6]. Enhanced recovery after surgery (ERAS) is a multimodal care pathway aiming to reduce perioperative stress response to decrease surgery-related morbidity. Among a multitude of measures aiming to simplify perioperative care, ERAS protocols endorse restrictive fluid management [7–11]. However, fluid management remains poorly defined and highly variable among institutions [12].

Postoperative fluid overload is a common problem in surgical patients, and physicians need reproducible, easy to monitor tools to prevent early complications and to guide further therapies. In clinical practice, daily fluid balances, diuresis, and body weight are commonly used to guide fluid management and diuretics therapy [13–15], but some studies showed that these two surveillance tools may disagree [16–18]. Considering the potential for calculation errors and imprecision with regard to insensible fluid losses, stool quantity etc., monitoring body weight may be more representative to identify fluid overload and associated postoperative complications.

Albumin is considered a negative acute-phase protein because its concentration decreases during injury and sepsis. The decrease of serum albumin was shown to be associated with postoperative outcomes and reflect extent of surgery [19–21]. Δ Albumin was used in the present study as a surrogate to correlate the impact of surgery to the fluid balances and weight evolution.

The aim of this study was to analyze daily weight variations and fluid balance during the first 3 postoperative days (PODs) after major surgery in a surgical intermediate care unit (IMC) of a high-volume institution and to correlate both measures to each other and postoperative outcomes. Whether one measure is preferable or more reliable than the other has not yet been assessed in the specific setting of a surgical intermediate care unit.

## Methods

Consecutive adult (≥ 18 years) patients undergoing elective or emergency major abdominal surgery (general anesthesia, > 2 h) with a request of direct postoperative surgical IMC at Lausanne University Hospital (CHUV) from 4 September 2017 to 30 January 2018 were eligible.

Patients hospitalized in external IMC wards outside the dedicated visceral surgery unit, patients remaining in the postanesthesia care unit (PACU) beyond the usual immediate postoperative surveillance period of 2 h, patients immediately admitted to the intensive care unit (ICU) for postoperative surveillance, and patients without informed consent for research participation were excluded. This is due to the data allocation and consistency with medical personal. In our institution, postoperative patients in the ICU and PACU are primarily managed by intensive care physicians and anesthetists, respectively, and therefore the patient’s weight protocols (i.e., timing, staffing, balance) are different. Patient admission to either ICU or IMC depends on multidisciplinary clinical evaluation postoperatively after the routine 2-h PACU surveillance period. Patients were admitted to the divisional surgical IMC (visceral surgery) based on this assessment.

This study was conducted as part of an institutional quality improvement project and data extraction was approved by the local Review Board (Commission cantonale d’éthique de la recherche sur l’être humain CER-VD # 2018-00249).

Demographic and surgical data of each patient were reported in a prospective database. Body mass index (BMI) and American Association of Anesthesiology (ASA) classification were calculated during preoperative anesthesiologic evaluation. Surgical details including duration and type of surgery were recorded. The duration of the procedure (from incision to closure) was recorded at the time of surgery either by the surgeon or the anesthetist in the institutional OR management software. All procedures were performed (either directly or under face-to-face supervision) by senior staff members of the respective surgical specialty.

Analysis of cumulative fluid balance, weight evolution, and composition of inputs/outputs were conducted only on patients with IMC stay of at least 72 h.

### Assessment of fluids and calculation of fluid balance

Intraoperative fluid balance was assessed and recorded by the anesthesiology care team. For elective surgeries, institutional surgical and anesthetic perioperative pathways, specific for each intervention and according to ERAS recommendations, were employed [7–11].

For emergency surgeries, perioperative fluid administration was guided by advanced hemodynamic monitoring (fluid guidance), hemoglobin (Hgb), acid bases status with periodic measurement of arterial blood gases, and urine output (> 0.5 ml/kg/h or up to discretion of treating anesthetist). If deemed necessary, invasive monitoring was used [18, 22].

The amount of intraoperative fluid administration was related to weight of patient, duration of surgery, blood loss, and urine output. Management was in line with recommendations of current consensus on perioperative fluid management [23–26]. Insensible losses were not taken into account for intraoperative fluid infusion [4, 7, 27].

Daily postoperative fluid balance was prospectively calculated according to the institutional protocol from 12 am to 12 am at PODs 1, 2, and 3. On a routine basis, the nursing staff in charge took into consideration all in/outputs. Continuous fluid perfusions, intravenous medications, epidural anesthesia/patient controlled anesthesia (PCA), nasogastric enteral feeding tube, oral intakes, and blood elements (packed red blood cells (pRBCs), fresh frozen plasma and platelets) were calculated and totaled for inputs. For output, calculation were considered gastric aspiration, vomiting, stool (if quantifiable), drains (if quantifiable through emptying, i.e., Jackson Pratt, intraperitoneal VAC, Penrose drain, chest tube, Redon drain), and urines. Insensible losses, such as perspiration, were not taken into account.

Transfusion thresholds for pRBCs were set in accordance with the institutional protocol: Hgb < 70 g/L in healthy, asymptomatic patients, Hgb < 90 g/L in polymorbid patients, particularly those with ischemic heart disease.

Daily weight was prospectively assessed on routine basis for all patients by the same standard scale every morning between 8 am and 12 am. In our institution, all elective patients are mobilized at POD 0, according to ERAS protocol routinely applied. The same strategy also applies to patients undergoing emergency surgery. This approach made it possible to weigh 86% of patients during the morning of POD 1. Difference of (Δ) weight was computed by comparing weight at PODs 1, 2, and 3 to preoperative body weight (assessed during preoperative outpatient visits within 30 days prior to surgery).

Serum albumin was routinely obtained in the setting of pre- and postoperative laboratory analyses. Δ albumin was assessed by comparing preoperative albumin (at POD − 1) to albumin at POD 1.

No institutional protocol for management of patients with fluid excess is currently available and diuretics administration was considered case-by-case and therefore not standardized.

### Outcomes/study endpoints

The primary outcome was the correlation between cumulative fluid balance (ml) and daily weight (kg) variations. More specifically, weight at POD 2 (previously identified as most predictive cutoff to launch potential counter-regulatory measures) was correlated with fluid balance at POD 1 (most accurately representing perioperative fluid balance) [28–30].

Secondary endpoint was the impact of positive cumulative fluid balance (ml) and weight gain (kg) on postoperative outcomes (complications and length of stay). Based on the results, cutoffs for the multinominal regression model were pragmatically set at 3 L (POD 1) and 3 kg (POD 2), respectively. Complications were graded according to the Clavien classification (grades I–V) [31] and the Comprehensive Complications Index (CCI) [32]. Two subanalyses were made to assess overall complications and major complications, as grades I to II were classified as minor and grades III to V as major, with grade V indicating death. Further outcomes of interest were length of IMC stay and length of hospital stay (LoS).

Outpatient control visits were routinely scheduled at 4–6 weeks from discharge.

Demographic and surgical characteristics were compared between two groups (< 24-h IMC stay vs. ≥ 24-h IMC stay) to identify patients needing solely overnight IMC surveillance.

### Subgroup analysis of patients with at least 72 h of IMC stay

To assess the impact of fluid balance and weight gain on postoperative outcomes, several subgroup analyses were performed in patients with IMC stay ≥ 72 h. In these patients, extent of surgery (Δ albumin, duration of surgery) was studied and correlated with cumulative fluid balance and Δ weight at POD 2 and postoperative morbidity (CCI).

Analyses were performed in the same group of patients to study the correlation between cumulative fluid balance, Δ weight and length of IMC/total length of stay at POD 2.

### Statistical analysis

Descriptive statistics were reported as median (interquartile range, IQR) and range or mean ± standard deviation (SD) as appropriate for continuous variables and absolute or relative frequencies for categorical variables. Continuous variables were compared using the Student *t* test; categorical variables through Fisher’s exact (chi squared) test. All tests were two-sided and *p* value of < 0.05 was considered statistically significant. Statistical correlations were tested by use of Pearson’s rank correlation.

Multinominal logistic regression was performed to compute odds ratios (OR) and 95% confidence intervals (CI) for the 4 outcomes any complication, major complication, prolonged IMC stay and prolonged total LoS, whereas prolonged IMC stay was defined as ≥ 3 days and prolonged LoS as ≥ 10 days (median total LoS for the whole cohort). For each multivariable model, all univariate risk factors with *p* < 0.1 for the respective outcome were included.

Data analysis was performed with the Statistical Software for the Social Sciences SPSS Advanced Statistics 22 (IBM Software Group, 200 W. Madison St., Chicago, IL; 60606 USA) and GraphPad Prism Software 8 (2365 Northside Dr., Suite 560, San Diego, CA; 92108 USA).

## Results

### Patients

**Fig. 1:**
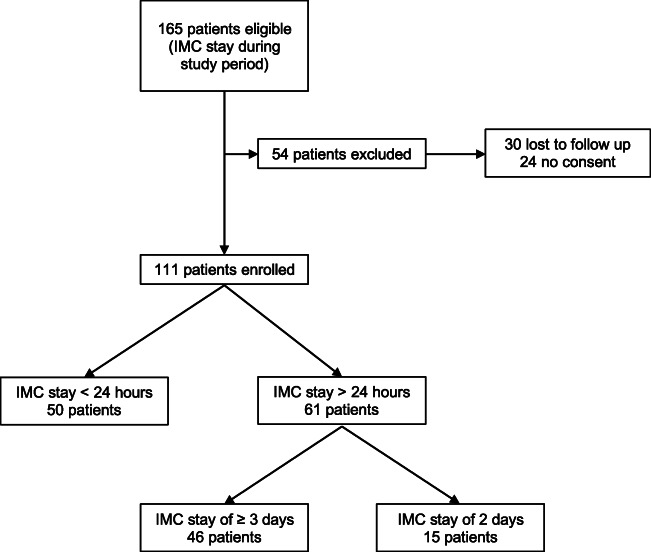
Study flow. Development of study cohort. IMC, surgical intermediate care unit

In total, 165 patients fulfilling the study requirements were admitted to IMC over the study period. Fifty-four patients (32.7%) were excluded for denial/lack of consent, as outlined in the flowchart in Fig. 1.

For the entire cohort, median cumulative fluid balance at the end of PODs 0–1–2–3 was 1850 (IQR 1020–2540) mL, 2890 (IQR 1610–4000) mL, 3890 (IQR 2570–5380) mL, and 4000 (IQR 1890–5760) mL respectively, and median weight gain was 2.2 (IQR 0.3–4.3) kg, 3 (1.5–4.7) kg, and 3.9 (2.5–5.4) kg, respectively.

Of the 111 included patients, 50 (45%) were discharged from IMC surveillance within 24 h, while 61 (55%) remained beyond POD 1. Demographic data of the cohort are displayed in Table 1. Patients in the longer surveillance group had a lower BMI and underwent more often HPB, open and prolonged procedures for malignant indications. Significant differences in early postoperative fluid balances and weight gain were observed between the 2 groups, as summarized in Table 2. Only 16 of the 111 patients (14%) were not weighed at POD1 and discharged from IMC surveillance before 12 AM.

**Table 1:** Demographics and surgical details

	All	Short IMC stay	Long IMC stay	
	(*n* = 111)	(*n* = 50)	(*n* = 61)	*p*
Age (years) (mean ± SD)	64 ± 13	64 ± 15	63 ± 11	0.685
Gender (male) (%)	68 (61)	31 (62)	37 (61)	1
BMI (kg/m^2^) (mean ± SD)	28 ± 8	31 ± 10	26 ± 5	0.003
ASA Group ≥ 3 (%)	60 (54)	29 (58)	31 (51)	0.45
Diabetes mellitus (%)	15 (14)	7 (14)	8 (13)	1
Heart disease (%)	22 (20)	10 (20)	12 (20)	1
Pulmonary disease (%)	14 (13)	6 (12)	8 (13)	1
WHO performance score ≥ 2 (%)	12 (11)	3 (6)	9 (15)	0.219
Preoperative albumin (g/L) (mean ± SD)	39.1 ± 6.7	40.3 ± 6.0	38.3 ± 7.2	0.189
Malignancy (%)	83 (75)	28 (56)	55 (90)	< 0.001
Open approach (%)	67 (60)	23 (46)	44 (72)	0.006
Emergency procedure (%)	15 (14)	8 (16)	7 (11)	0.581
Procedure group				0.028
Hepatobiliary (%)	25 (23)	8 (16)	17 (28)	
Pancreas (%)	19 (17)	5 (10)	14 (23)	
Colorectal (%)	19 (17)	8 (16)	11 (18)	
Oesogastric (%)	22 (20)	11 (22)	11 (18)	
Other (%)	26 (13)	18 (36)	8 (13)	
Operation duration (min) (mean ± SD)	240 ± 120	190 ± 110	270 ± 120	< 0.001
Operation duration > 270 min (%)	44 (40)	12 (24)	32 (52)	0.003

Baseline demographic and surgical parameters of patients with short IMC stay of ≤ 24 h (*n* = 50) and patients with long IMC stay of > 24 h (*n* = 61)

*IMC* Surgical intermediate care unit, *BMI* body mass index, *ASA* American Society of Anesthesiology, *WHO* World Health Organization

**Table 2:** Fluid-management related parameter

	All	Short IMC stay	Long IMC stay	
	(*n* = 111)	(*n* = 50)	(*n* = 61)	*p*
Fluid balance POD 0 (mL) (mean ± SD)	1850 ± 1200	1530 ± 980	2120 ± 1310	*0.007*
Fluid balance POD 1 (mL) (mean ± SD)	900 ± 930	720 ± 820	990 ± 920	0.169
Δ weight POD 1 (kg) (mean ± SD)	2.2 ± 3.1	1.4 ± 2.8	2.8 ± 3.1	*0.023*
Δ weight POD 2 (kg) (mean ± SD)	3.0 ± 3.0	1.3 ± 3.1	3.6 ± 2.7	*0.003*
Δ weight POD 3 (kg) (mean ± SD)	2.7 ± 2.8	0.6 ± 3.0	3.9 ± 2.6	*0.001*
Δ albumin POD 1 (g/L) (mean ± SD)	7.6 ± 5.7	6 ± 5.2	8.5 ± 5.9	0.062

Fluid-management related parameter of patients with short IMC stay of ≤ 24 h (*n* = 50) and patients with long IMC stay of > 24 h (*n* = 61)

*POD* Postoperative day, *Δ* difference, *IMC* surgical intermediate care unit

### Subgroup analysis of patients with ≥ 3 days of IMC stay

**Fig. 2:**
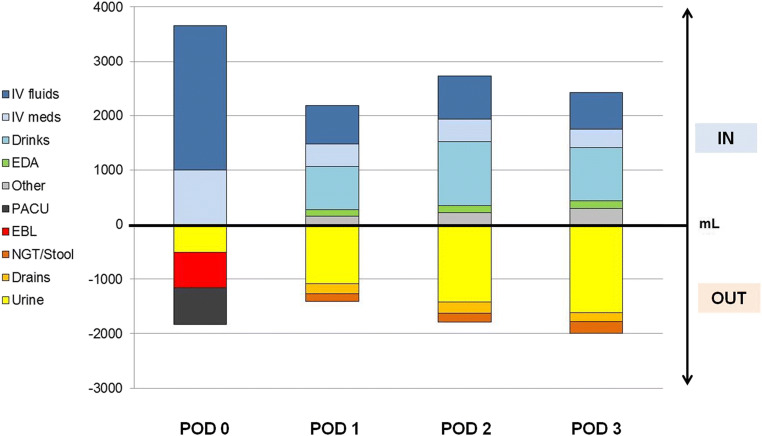
Detailed fluid balance during IMC stay. Detailed representation of fluid balance and composition of administered (IN) and excreted (OUT) fluids through PODs 0–3 in patients with Surgical Intermediate care unit (IMC) stay of at least 3 days (*n* = 46). Inputs consider IV fluid drips (dark blue), IV drugs (light blue), clear oral liquids (turquois), EDA/PCA (green), and other (enteral tube feeding, blood elements, flushes > 10 mL). Outputs consider urine (yellow), estimated blood loss (red), charted losses during IMC stay (black), surgical drain output (orange) and NGT/vomiting/stool (if quantifiable) output (brown). IV—intravenous, EDA—epidural anesthesia, PCA—patient controlled anesthesia, IMC—surgical intermediate care unit, NGT—nasogastric tube, POD—postoperative day

Forty-six patients (41%) remained in the IMC for at least 3 days, and all of them were weighed until 12 AM, according to study protocol. Detailed composition of fluid administration and fluid losses are displayed in Fig. 2.

**Fig. 3:**
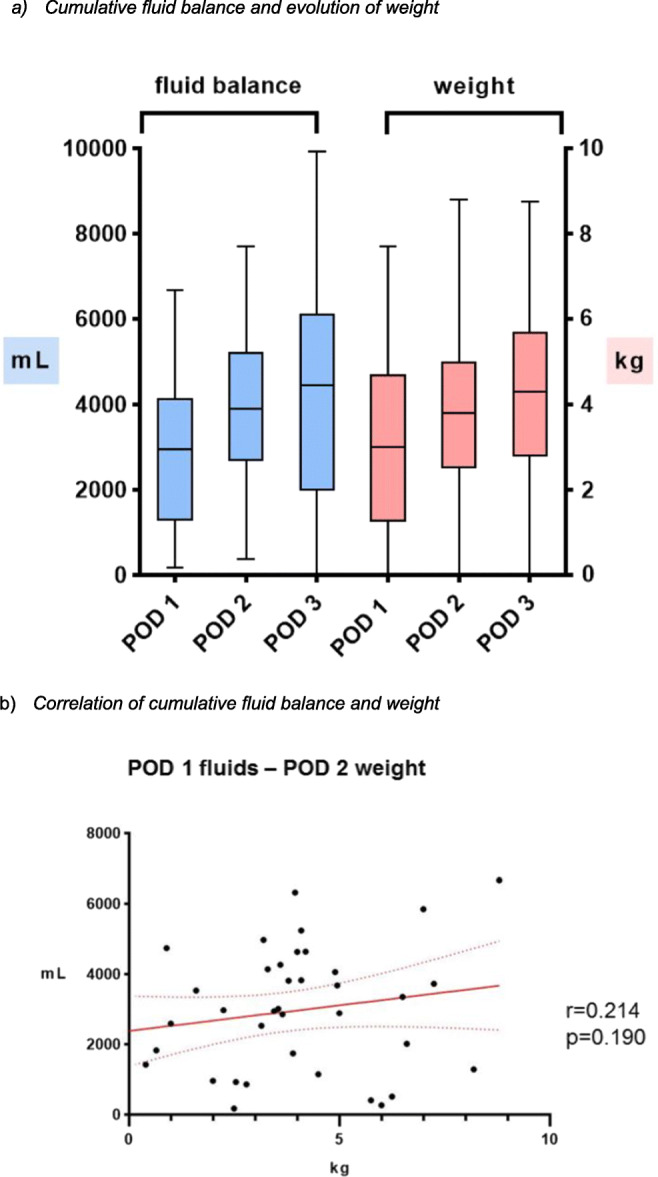
Cumulative fluid balance and evolution of weight and correlation of cumulative fluid balance and weight. **a** Cumulative fluid balance and evolution of weight. Whisker plots displaying cumulative fluid balance (blue) and evolution of weight (red) through PODs 1–3 in patients with IMC stay of at least 3 days (*n* = 46). POD—postoperative day, mL—milliliter, kg—kilogram, IMC—surgical intermediate care unit. **b** Correlation of cumulative fluid balance and weight. Correlation of cumulative fluid balance at POD 1 and weight at POD 2. mL—milliliter, kg—kilogram

Figure 3a outlines cumulative fluid balances and weight evolution (Δ weight) from the day of surgery through POD 3, showing almost identical patterns. Figure 3b shows no correlation of cumulative fluid balance at POD 1 with weight at POD 2 (*p* = 0.190).

**Fig. 4:**
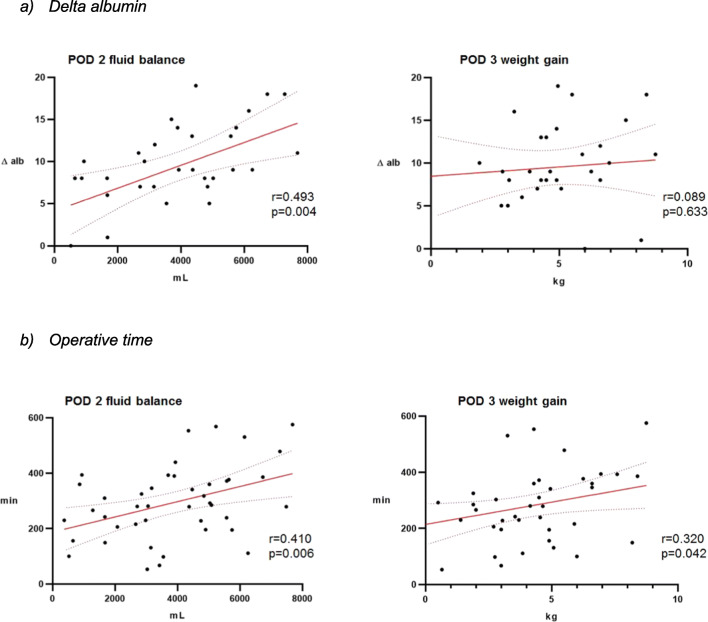
Impact of extent of surgery on fluid balance and weight. **a** Delta albumin. **b** Operative time. Correlation of extent of surgery through surrogates **a** Δ-albumin and **b** operative time and cumulative fluid balance at POD 2 and weight gain at POD 3 in patients with IMC stay of at least 3 days (*n* = 46). POD—postoperative day, Δ alb—Δ albumin, min—minutes, *r* = Pearson correlation coefficient, *p* < 0.05 is considered statistically significant, IMC—surgical intermediate care unit

Extent of surgery (as assessed through surrogates Δ albumin and surgical duration) correlated significantly with POD 2 fluid balances, as illustrated in Fig. 4 a and b. While POD 3 weight gain correlated with surgical duration, no significant correlations were observed between POD 3 weight gain and Δ albumin.

**Fig. 5:**
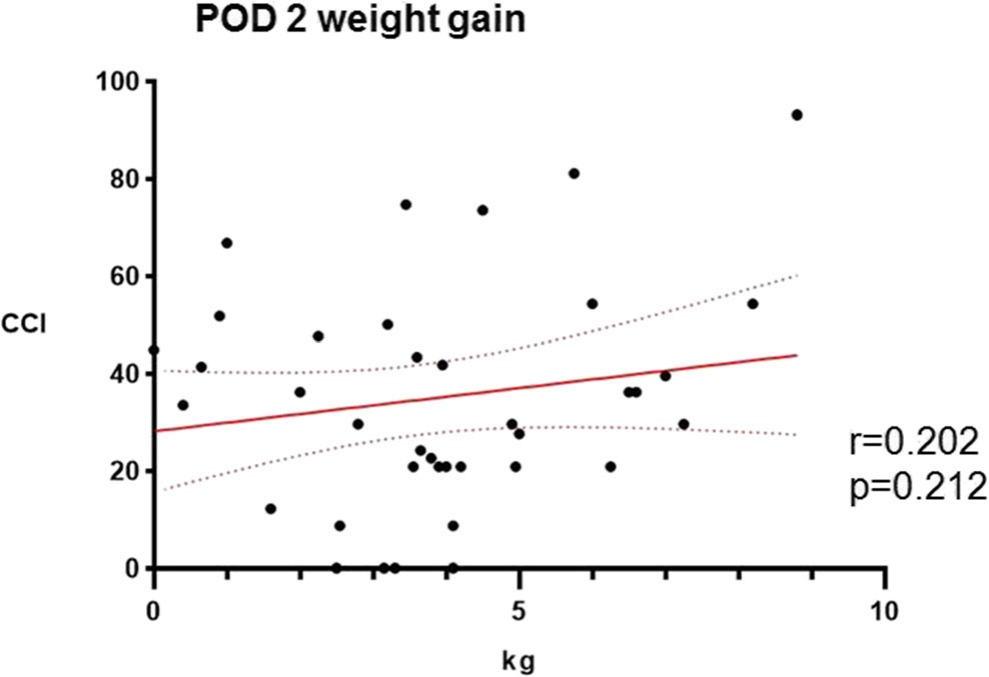
Impact of weight gain on postoperative morbidity. Correlation of the comprehensive complication index (CCI) and weight gain at POD 2 in patients with IMC stay of at least 3 days (*n* = 46). POD—postoperative day, IMC—surgical intermediate care unit. *r* = Pearson correlation coefficient, *p* < 0.05 is considered statistically significant

POD 2 weight gain did not significantly correlate with surgical morbidity as assessed by the CCI as shown in Fig. 5.

A total of 13/46 patients (28%) with ≥ 3-day IMC stay experienced major complications. Regression analysis was not performed (small sample size/event rate).

**Fig. 6:**
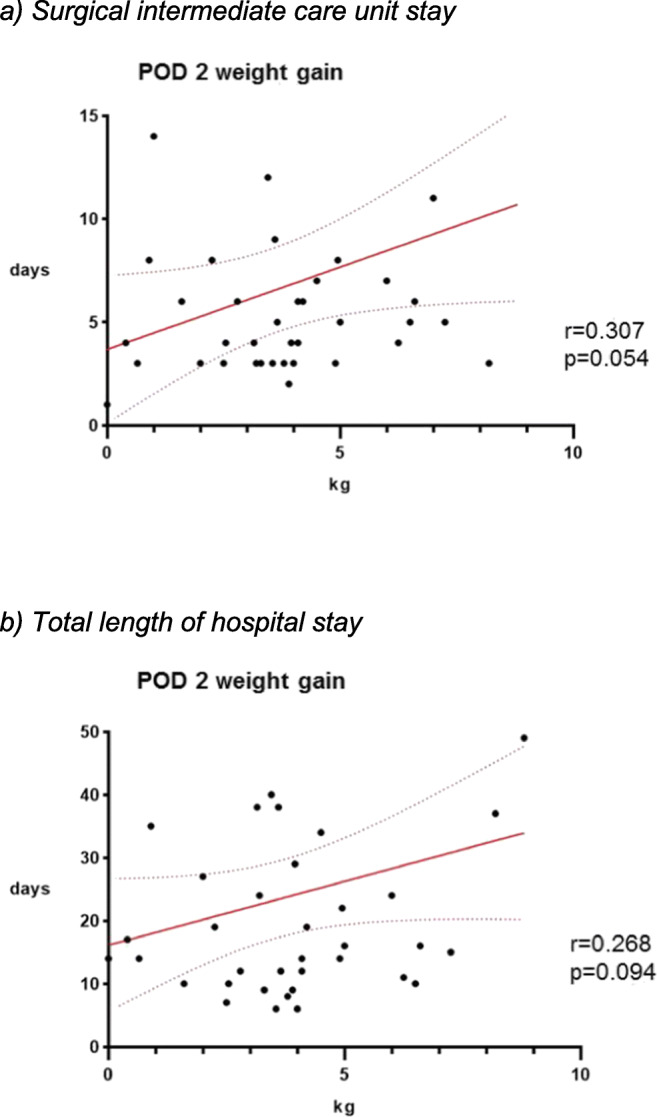
Impact of weight gain on length of stay. **a** Surgical intermediate care unit stay. **b** Total length of hospital stay. Correlation of length of intermediate care unit stay and total length of hospital stay and weight gain at POD 2 in patients with IMC stay of at least 3 days (*n* = 46). POD—postoperative day, IMC—surgical intermediate care unit. *r* = Pearson correlation coefficient, *p* < 0.05 is considered statistically significant

Figure 6 correlates POD 2 weight gain to IMC stay (*p* = 0.054) and total hospital stay (*p* = 0.094) in the subgroup of patients staying at least 3 days.

Seventy-five patients (66%) experienced any complication, while 34 (31%) presented a major complication. Median IMC stay for all patients was 3 days (IQR 1–5 days), while median total LoS was 10 days (IQR 6–17 days).

The only independent risk factor for any complication was open surgery (OR 3.08, 95% CI 1.02–9.24, *p* = 0.045), while open surgery (OR 3.37, 95% CI 1.02–11.08, *p* = 0.046) and preoperative hypoalbuminemia (OR 5.54, 95% CI 1.12–27.5, *p* = 0.036) were identified as risk factors for major complications. Prolonged IMC stay of ≥ 3 days was related to weight gain ≥ 3 kg at POD 2 (OR 2.8, 95% CI 1.01–8.9, *p* = 0.049), while prolonged total LoS was independently associated with open surgery (OR 2.98, 95% CI 1.22–7.28, *p* = 0.017), malignancy (OR 5.96, 95% CI 1.98–17.95, *p* = 0.001), and emergency surgery (OR 5.01, 95% CI 1.14–22.05, *p* = 0.033). Detailed results of multivariable analysis are displayed in online appendix 1.

## Discussion

This retrospective analysis showed a weak correlation between postoperative fluid balance and weight change. No correlation was found between postoperative weight gain and complications. However, increasing weight gain positively correlated with prolonged length of hospital stay.

In clinical practice, both daily fluid balance and body weight are used to guide fluid therapy [13–15]. Whether the more complex and detailed calculation of fluid balance or simply postoperative weight evolution is more predictive remains matter of debate. Calculation of fluid balance is complex, imprecise, and can be prone to systematic error. Considering the potential for errors due to insensible fluid losses when calculating fluid balance, the objective, and reproducible weight-based method may be more representative. Obtaining a reliable body weight measurement requires compliance by nursing staff, which is, sometimes, difficult for the workload and patient’s clinical presentation, especially for hemodynamic condition. In our experience, only 14% of patients were not weighed at POD 1, but all of them were discharged from IMC before 12 h, and all patients were weighted at POD3. These results could be explained by the routine use of the ERAS protocol since several years, which focuses particularly on early postoperative mobilization of the patient already at the day of surgery, also if hemodynamic support is needed. In the present study, the correlation between cumulative fluid balance and weight gain in the first three postoperative days was weak. As illustrated in Fig. 3a, the evolution of cumulative fluid balance appears to be almost identical to weight gain through PODs 1–3, and Fig. 3b shows a low correlation between fluid balance at POD 1 and weight at POD 2. This can be explained by limitations related to the small sample size. Furthermore, these findings suggest that daily weight may be a simpler and more objective way to measure postoperative fluid shifts. Several studies have reported a lack of accuracy in calculating fluid balances. Perren et al. [33], in a study of 147 patients carried out in ICU, revealed that daily and cumulative fluid balance were arithmetically incorrect in one third of cases. Similarly, Köster et al. [16] found that cumulative daily fluid balances did not correspond with weight changes in 106 ICU patients with length of ICU stay > 5 days, even after consideration of insensible fluid losses. Tolstrup et al. [17] revealed a significant discrepancy between the two techniques on postoperative day 5 (> 2000 g/mL).

In the present study, positive fluid balance reflected the extent of surgery (duration, Δ albumin). This is in line with recent studies stressing the relationship between surgical stress and postoperative albumin decrease. Mantziari et al. [34] reported on 70 patients undergoing seven different surgical interventions to show a correlation between surgical stress and biomarkers (CRP, albumin, and triglycerides). However, only albumin changes (delta albumin) correlated with surgical access, peritoneal trauma, and organ resections. Lagbaa et al. [19] analyzed 138 patients undergoing major surgery and found a correlation between Δ albumin and surgical stress. Further, the decrease of serum albumin was closely associated with postoperative adverse outcomes in their analysis.

As illustrated in Fig. 4, the findings of the present study show a significant correlation between POD 2 fluid balance and both postoperative albumin decrease and operative time. This can be explained by the surgical stress response, which induces inflammatory and hormonal perturbation, which in consequence impacts on salt and water metabolism [35]. Furthermore, a tendency to normalization of weight at POD 3 as seen in Fig. 4 was observed. This may reflect, besides the end of the early post-operative inflammatory response, early triggering of counter-regulatory measures (i.e. diuretic therapy) and interruption of IV infusion routinely initiated during surgery, in line with the ERAS protocol [7–11, 36]. Similar findings were described by Tolstrup et al. [17]: in their study, patient’s weight decreased after POD3 while discrepancy between body weight and fluid balance increased.

The population of the present study was separated in two groups, based on the length of IMC stay. The two groups differed regarding surgical details (malignancy, open approach, operation duration), but also BMI. This BMI difference can be explained by the institutional policy to systematically monitor bariatric patients with untreated sleeping apnea for 24 h in IMC (12 patients, 10.8%).

The association between highly positive fluid balance and poor outcome has been previously observed. Several studies reported that positive fluid balance may result in worse outcome and prolonged length of stay [5, 8]. Köster et al. [16] confirmed these results and showed increased survival and reduced ICU stay in all comers experiencing weight loss of 1.8 kg at the time of discharge. Our group previously studied effects of fluid balance on postoperative outcomes after ileostomy takedown. Both excess fluids and weight gain were found to be independent predictors of postoperative ileus and emphasize the importance of stringent fluid management [6].

Weinberg et al. [37] studied 150 patients after Whipple’s procedure and found better outcomes in terms of length of hospital stay and postoperative complications in patients with restrictive fluid management.

The present study further suggests that excessive weight gain may be associated with prolonged IMC and hospital stay, but our observations need to be confirmed by adequately powered studies, as seen in Fig. 6.

Postoperative complications were more common after open surgery and in malnourished patients as shown in Table 1, while no significant association with weight gain was observed as displayed in Fig. 5. Potential explanations include routine use of ERAS protocols in our institution, with implementation of a wide array of measures to counteract positive fluid balances early in the postoperative course, including IV fluid lock at POD1 and both early resumption of oral intake and mobilization [7–11, 36].

This study has several limitations. The modest simple size makes the study prone to type II error, and larger studies are needed to confirm these preliminary data. This pilot study may be underpowered for some of the secondary outcomes, which were only assessed in the subgroup of patients with ≥ 3 days of IMC stay. This has to be considered when interpreting the results, and future prospective studies with predefined, adequate sample sizes for the respective outcome of interest are mandatory. Furthermore, the specific setting of a surgical IMC unit potentially represents a selection bias and thus the results of the present study cannot be uncritically extrapolated to other care settings (i.e., ICU, general wards). The study is further limited by its retrospective design, despite thorough prospective data gathering. Arguably, the study cohort is heterogeneous, however reflecting the diverse surgical activity of the present institution.

## Conclusion

The present study suggests that postoperative weight evolution may be sufficient as surveillance tool to predict complications in the early postoperative course. Larger studies are needed to confirm these results.

## Electronic supplementary material

ESM 1 (DOCX 16 kb)

## Abbreviations

ICU: Intensive care unit
IMC: Surgical intermediate care unit
ERAS: Enhanced recovery after surgery
POD: Postoperative day
PACU: Postanesthesia care unit
pRBCs: Packed red blood cells
PCA: Anesthesia/patient controlled anesthesia
CCI: Comprehensive Complications Index
