# Supplementary material for: Correlation of postoperative fluid balance and weight and their impact on outcomes
Source: Langenbecks Arch Surg. 2020 Oct 13;405(8):1191–200. doi: 10.1007/s00423-020-02004-9 (PMC7686193; doi:10.1007/s00423-020-02004-9)
Supplement: Supplementary file 1 — (DOCX 16 kb) [file 423_2020_2004_MOESM1_ESM.docx]

# Online Appendix 1 Multivariable analysis

|  | **Any complication** | **Major complication** | **Prolonged IMC stay** | **Prolonged LoS > 10 days** |
| --- | --- | --- | --- | --- |
| **Male sex** | 2.29 (0.78-6.76) | - | - | - |
| **Open surgery** | **3.08 (1.02-9.24)** | **3.37 (1.02-11.08)** | 2.34 (0.74-7.43) | **2.98 (1.22-7.28)** |
| **Hypoalbuminemia** | - | **5.54 (1.12-27.5)** | - | - |
| **Duration > 270 min** | 2.82 (0.86-9.21) | - | 1.67 (0.52-5.39) | 1.82 (0.74-4.50) |
| **Malignancy** | - | - | 3.63 (0.89-14.86) | **5.96 (1.98-17.95)** |
| **Emergency** | - | 2.65 (0.54-13.1) | - | **5.01 (1.14-22.05)** |
| **WHO score ≥ 2** | 7.4 (0.77-20.2) | - | - |  |
| **Weight gain** |  |  |  |  |
| > 3 kg POD 2 | - | - | **2.8 (1.01-8.9)** | 1.54 (0.82-4.43) |
| **Fluid balance** |  |  |  |  |
| > 3 L POD 1 | 2.16 (0.73-6.39) | - | - | - |

Multivariable analysis of 4 different outcomes any complication, major complication (Clavien ≥ III), prolonged IMC stay (≥ 3 days) and total hospital stay > 10 days. All univariate risk factors (p<0.1) for each outcome were retained. Displayed are Odds ratio and 95% confidence interval. Bold characters indicate significant values.

IMC – Intermediate Care Unit, WHO – World Health Organization performance score, POD – postoperative day.
